# Oblique scanning laser microscopy for simultaneously volumetric structural and molecular imaging using only one raster scan

**DOI:** 10.1038/s41598-017-08822-0

**Published:** 2017-08-17

**Authors:** Lei Zhang, Amalia Capilla, Weiye Song, Gustavo Mostoslavsky, Ji Yi

**Affiliations:** 1 Department of Medicine, Boston University School of Medicine, Boston, MA 02118 USA; 2 Center of Regenerative Medicine, Boston University, Boston, MA 02118 USA; 3 Boston University Photonics Center, Boston, MA 02215 USA

## Abstract

Multi-modal three dimensional (3D) optical imaging combining both structural sensitivity and molecular specificity is highly desirable in biomedical research. In this paper, we present a method termed oblique scanning laser microscopy (OSLM) to combine optical coherence tomography (OCT), for simultaneously volumetric structural and molecular imaging with cellular resolution in all three dimensions. Conventional 3D laser scanning fluorescence microscopy requires repeated optical sectioning to create z-stacks in depth. Here, the use of an obliquely scanning laser eliminates the z-stacking process, then allows highly efficient 3D OCT and fluorescence imaging by using only one raster scan. The current setup provides ~3.6 × 4.2 × 6.5 μm resolution in fluorescence imaging, ~7 × 7 × 3.5 μm in OCT in three dimensions, and the current speed of imaging is up to 100 frames per second (fps) over a volume about 0.8 × 1 × 0.5 mm^3^. We demonstrate several mechanisms for molecular imaging, including intrinsically expressed GFP fluorescence, autofluorescence from Flavin proteins, and exogenous antibody-conjugated dyes. We also demonstrate potential applications in imaging human intestinal organoids (HIOs), colon mucosa, and retina.

## Introduction

Volumetric optical imaging with cellular and sub-cellular resolution is essential for our fundamental understanding of biological systems. One critical aspect of optical imaging is the 3D localization of molecular composites in tissues or cells, typically by using specific antibodies with fluorescent reporters. However, this molecular specificity comes with a dilemma that the structural context and other unspecified molecules would not appear in the images, *i.e*. we can only see what we choose to see. This missing information can confuse and sometimes mislead our interpretation. For example, the detection of fluorescence not only depends on the absolute amount of targeted molecules, but also the local structural density which can limit the diffusion and binding of the antibodies. Therefore, there is an increasing need for multimodal imaging techniques that can provide both molecular specificity and the structural context. Despite a large body of literatures and long standing efforts on this subject, it is still challenging to provide a multimodal system that can achieve volumetric structural and molecular imaging simultaneously with equivalent high resolutions for both modalities.

Early work on multi-modal 3D optical imaging is based on confocal laser scanning microscopy (LSM), where a confocal pinhole is applied for the depth sectioning and a stack of transverse images are taken for 3D reconstruction^1^. In addition to the fluorescence mode for molecular specificity, LSM can simultaneously detect the backscattered light in the reflectance mode which is sensitive to structural heterogeneity^2, 3^. Since the development of LSM, a variety of different microscopic techniques have been adopted to improve the multimodal capabilities for either fluorescence or reflectance modes. For instance, multi-photon microscopy has been used for the fluorescence mode to extend the depth penetration in highly scattering medium and to allow label-free molecular contrast^4–6^. For the reflectance mode, the confocal gating has been replaced with coherence gating to improve the depth resolution^4–6^. A combination of coherence gating and structured illumination allows a 3D multimodal imaging under a wide field microscopy with only incoherent sources^7^. However, the common and major limitation of these imaging methods is that the depth sectioning primarily depends on the focusing power of the objective lens, which leads to practical limitations on depth penetration, unequal lateral and depth resolution, photo bleaching and imaging speed. For example, to obtain high depth resolution, an objective lens with high numerical aperture (NA) and a dense depth sampling are required, which inevitably limit the field of view (FOV) in each acquisition.

To address these limitations, light sheet fluorescence microscopy (also called selective plane illumination microscopy) has been developed to decouple the depth resolution from the objective lens and use a second orthogonally aligned objective lens to provide a “light of sheet” for depth sectioning^8^. This allowed a high throughput volumetric imaging system with sub-cellular resolution over 1 × 1 × 1 mm^3^ within several seconds^9^. It was later demonstrated that the light sheet microscopy can be implemented with a single objective lens, a method termed oblique plane microscopy (OPM)^10^, to achieve video rate 3D imaging at a microscopic volume^11, 12^. More recently, the full advantage of OPM has been demonstrated by swept confocally aligned planar-excitation microscope (SCAPE)^13^, which achieved high speed volumetric imaging on a macroscopic volume of mouse cortex *in vivo* at 10 Hz and freely moving *Drosophila* larvae at 20 Hz. However, there is not yet a multimodal system that can simultaneously provide structural imaging co-registered with light sheet fluorescence microscopy. Optical projection tomography (OPT) has been integrated to image the structural contrast^14^. The limitation is that OPT and light sheet microscopy were operated separately, and the transmission configuration of OPT significantly limits the *in vivo* applications.

On the other hand, optical coherence tomography (OCT) has emerged to be an important volumetric structural imaging modality capable of micron/sub-micron level resolution and up to several millimeter depth of penetration *in vivo*. Regardless of its lack of molecular specificity, OCT has exquisite sensitivity to structural changes even at sub-diffractional length scales (several tens of nanometers) without actually resolving the detail nanoarchitecture^15, 16^. In addition, OCT can provide comprehensive measurements of blood flow^17, 18^, blood oxygenation^19, 20^ and capillary-level angiography^21, 22^, altogether making it a powerful tool to characterize the microenvironment in living tissues. Since OCT also adapts the laser scanning scheme, it has been integrated with LSM in various forms, such as in a conventional microscopic LSM setting^4–7, 23–26^, in an endoscopic form with fluorescence contrast agent or autofluorescence^27–30^, and in the ophthalmic imaging systems^31, 32^. However, none of these approaches provides simultaneous 3D imaging of both OCT and fluorescence due to their different depth discrimination mechanisms. The depth sectioning in OCT relies on the coherence gating by interfering the scattered light with a reference light, in such a way that 3D imaging can be implemented by only one raster scan of the laser. This is in contrast with the repeated raster scans used in LSM for 3D imaging. Aside from LSM, fluorescence laminar optical tomography (FLOT) has been used for a multimodal imaging system with OCT^33, 34^. The caveat there is that FLOT is based on a diffusive contrast which limits the resolution to several hundreds of microns.

In this paper, we present a multimodal volumetric imaging system implementing a method that we called oblique scanning laser microscopy (OSLM). Combined with OCT, the system is capable of simultaneous structural and molecular imaging with cellular resolution with only one raster scan on the sample. The system adopted the oblique illumination scheme with a single objective lens as in OPM and SCAPE. By reducing the 2D light sheet illumination to a 1D laser scanning, OCT can be seamlessly and simultaneously incorporated and provide structural imaging with equivalent resolutions. The current setup provided ~3.6 × 4.2 × 6.5 μm resolution in OSLM, ~7 × 7 × 3.5 μm in OCT in three dimensions, and a volumetric FOV around ~0.8 × 1 × 0.5 mm^3^. In organizing this paper, we firstly introduced the system setup and methods for our experiments, then provided the characterization of OSLM, and several examples of potential applications including imaging intestinal organoids differentiated from genetically modified human induced pluripotent stem cells (iPSC), intrinsic fluorescence from colonic mucosa, and mouse retina with stained endothelium. In the end, we discussed the limitation of the current system and potential *in vivo* applications.

## Methods and Materials

All animal procedures were approved by the Institutional Animal Care and Use Committee at Boston Medical Center and conformed to the guidelines on the Use of Animals from the National Institutes of Health (NIH).

### System setup

**Figure 1:**
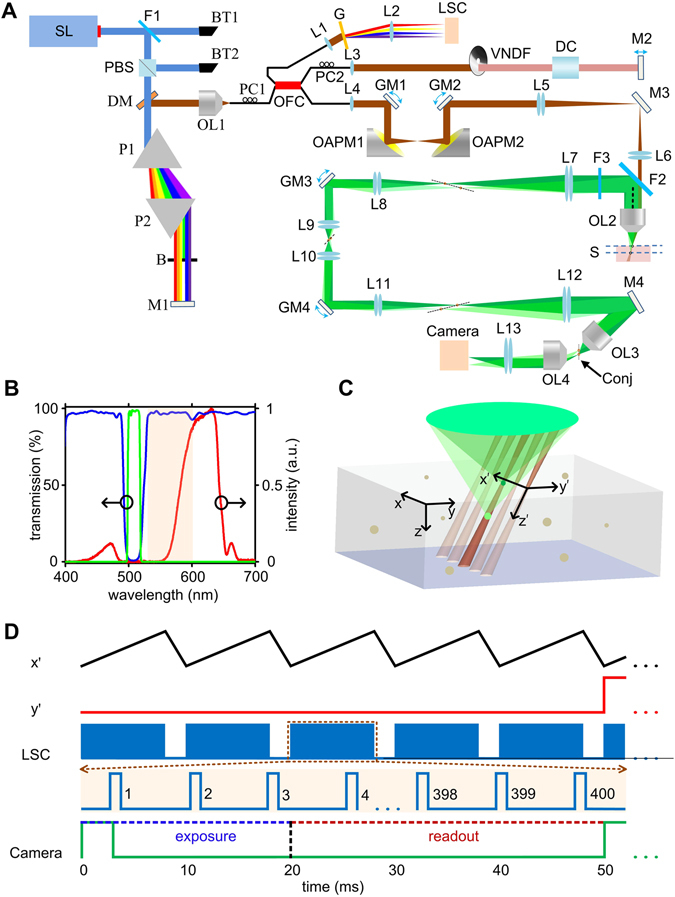
System design of the oblique scanning laser microscopy (OSLM). (**A**) The schematic of OSLM system. SL: supercontinuum laser; F: filter; BT: beam trap; PBS: polarization beam splitter; P: prism; B: block; M: mirror; DM: D-shaped mirror; OL: objective lens; PC: polarization controller; OFC: optical fibre coupler; L: lens; VNDF: variable neutral density filter; DC: dispersion compensator; G: grating; LSC: line scan camera; GM: galvano mirror; OAPM: off-axis parabolic mirror; S: sample. Conj: the conjugate space to the sample space. (**B**) The optical spectra of the illumination (red), and the filter transmission spectra for the dichroic mirror F2 (blue) and filter F3 (green). The shaded pink area indicates the collection range of the OCT spectrometer. (**C**) The 3D model of OSLM at the sample space. The dimensions of the laser scanning are *x’* (fast scanning), *y’* (slow scanning) and *z’* (depth). The geometric dimensions are denoted as *x, y, z* for a comparison. (**D**) The control signals for the galvanometer scanning mirrors and the cameras. For LSC, each blue block contains 400 triggers for OCT B-scan acquisition. For the CCD camera, the green curve is the trigger signal to start the exposure. The voltage is scaled and reversed for the de-scanning galvanometer mirrors.

The system schematic is shown in Fig. 1A. A supercontinuum laser (SL, SuperK, NKT photonics) was used to generate the broad band laser output. The visible portion (420–650 nm) was filtered out using a dichroic mirror, and polarized by a polarization beam splitter (PBS). A pair of prisms dispersed the spectrum onto a reflecting mirror (M1), such that a thin aluminum film can be inserted to block the fluorescence emission band. Figure 1B shows the illumination spectrum, where the 420–470 nm range excited the fluorescence and the range above 550 nm was used for OCT. The light is coupled into a 50/50 optical fibre coupler (OFC), and collimated by an *f* = 4.5 mm lens. Two galvanometer scanning mirrors (GM1, GM2) and two parabolic mirrors were mounted on a custom-made raster scanning unit to steer the laser. Then a telescope system (L5 + L6) relayed the beam to the back pupil of the objective (Olympus UPLAN NA 0.5). The optical axis of the telescope system (L5, L6) and that of the objective was offset to create oblique laser illumination. The objective and a double band dichroic mirror (ZT514/1064rpc, Chroma) were mounted on a custom-made dove tail slider to adjust the offset and the oblique angle. An offset of 4 mm resulted in a ~26° of angle with respect to the optical axis of the objective (Supplemental Fig. 1).

The fluorescence light was redirected by the dichroic mirror and further filtered by a band-pass filter. Three 4-f telescope systems (L7 + L8, L9 + L10, L11 + L12) were used to relay the light from the back pupil plane of OL2 to two de-scanning galvanometer mirrors (GM3, GM4) and finally, to the back pupil of the second objective lens (OL3). To reduce the spherical aberration and astigmatism, pairs of identical achromatic doublets were used for all the relay optics (L5-L12)^35^. The magnification from the sample plane in front of OL2 to the image plane after OL3 was ~2/3 and the angle of the image was magnified to ~40° with respect to the optical axis of OL3 Supplemental Fig. 2). The choice of this magnification is to have a proper angle of image after OL3, so that we can balance the light collection efficiency from OL3 to OL4 and a moderate focal length of L13 (*f* = 50 mm in current setup). The calculation of the angle of image with respect to the magnification is described in the supplemental materials. Finally, another imaging system (OL4 + L13) was aligned in an angle of around 50° to refocus the angled image on to a CCD camera (PCO Pixelfly-USB).

For OCT, the reference beam was collimated and reflected by a mirror (M2). A variable ND filter and several BK7 glass plates (DC) were installed in the reference arm to attenuate the light, and to compensate the dispersion in the sample arm. The returning light was sent to a custom-made spectrometer. The detailed part components are included in the supplementary materials.

The 3D model of OSLM at the sample space is shown in Fig. 1C, where the 1D scanning laser illuminates an oblique plane *x’*-*z’* by the fast scanning, and the volumetric imaging is achieved by another slow scan in *y’*. The tilted image of *x’-z’* at the conjugate space after OL3 were then refocused onto the CCD camera.

### System synchronization

The synchronization sequence for our system is illustrated in Fig. 1D. The fast galvanometer scanning mirror in *x’* direction was controlled by a saw tooth voltage with an 80% duty cycle. Five repeated scans were given at each slow axis in *y’* direction. In each forward scanning direction in *x’*, 400 triggers were sent to the line scan camera for OCT acquisition. At each *y’* location, a single trigger was given to the 2D CCD camera to start the exposure. The exposure time was maximized to just allow sufficient time for data transfer before the next frame. The galvo driving signals and the triggers were generated from an analogue output (AO) card (PCI-6731, National Instruments). For the de-scanning mirrors, we reversed and scaled the waveforms for the scanning mirrors and sent the voltage from two AO ports of the DAQ card (PCIe-6351, National Instruments). When using the CCD camera, only one of the de-scanning mirror was synchronized with the slow axis scanning mirror and the second de-scanning mirror was kept still. Unless otherwise specified, the OCT A-line rate was 50 kHz. The 2D CCD camera exposure time is 20 ms. The maximum frame rate is 100 fps for OCT, 20 fps for camera.

### Numerical simulation for resolution characterization

**Figure 2:**
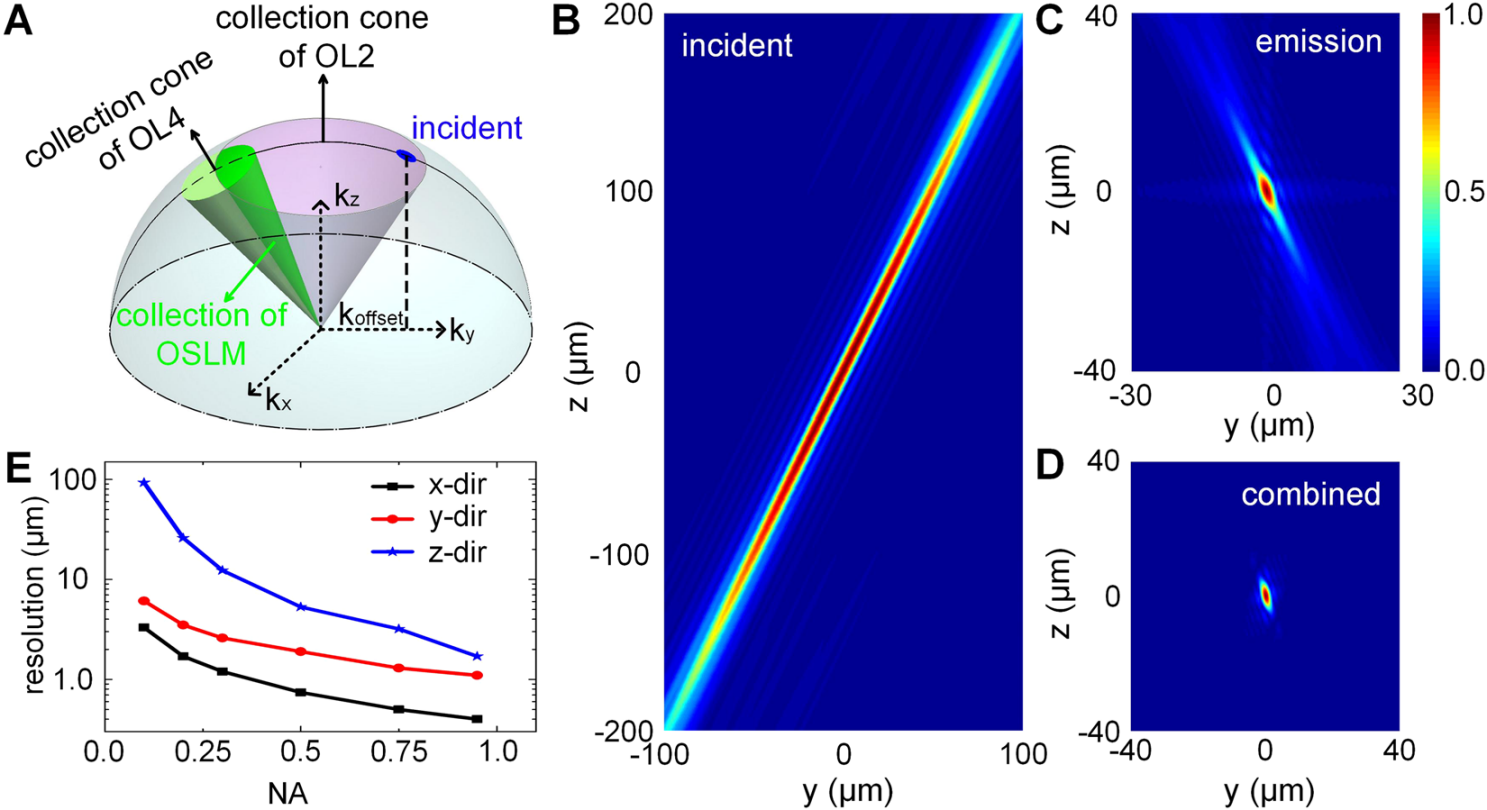
The Fourier domain model of the oblique scanning laser microscopy (OSLM). (**A**) The range of the incidence and detection in the 3D reciprocal Fourier domain. The blue area on the spherical dome represents the illumination, and the overlapping area projected onto the dome from two collection cones of OL2 and OL4 shows the detectable spatial frequency range by OSLM. (**B**) The incident, (**C**) emission and (**D**) combined point spread functions (PSFs) of the system. (**E**) The theoretical resolutions in three directions under various numerical aperture of the objective lens, under the same detection range in (**A**). The resolution is measured by FWHM crossing the center of the 3D PSF in panel D.

The schematic of the simulation is shown in Fig. 2A. A 3D matrix in Fourier space was firstly created with initial value of zeros. The coordinate was defined by a wave vector

$${\boldsymbol{k}}=\frac{2\pi }{\lambda }\hat{k}=({k}_{x},{k}_{y},{k}_{z})\,,$$

where $\hat{k}$ is the directional vector with unit magnitude. The range of three orthogonal directions were −2*π/λ* < *k*_*x*_ < *2π/λ*, −*2π/λ* < *k*_*y*_ < *2π/λ*, and 0 < *k*_*z*_ < *2π/λ*. The wavelength λ was set to be 500 nm for simplicity. Next, the spatial frequency range corresponding to the illumination was identified: (−*2π/λ*) × NA_i_ < *k*_*x*_ < (*2π/λ*) × NA_i_, (−*2π/λ*) × NA_i_ < *k*_*y*_ − *k*_*offset*_ < (*2π/λ)* × NA_i_, and *k*_*z*_^2^ + *k*_*x*_^2^ + *k*_*y*_^2^ = (*2π/λ*)^*2*^, as the blue area on the dome in Fig. 2A. The NA_i_ is the numerical aperture of the illumination which is set to be 0.04 according to the experimental setup. The laser incidence is offset in Fourier domain from *k*_*z*_ axis by *k*_*offset*_ for the oblique illumination, as shown in Fig. 2A.

For fluorescence emission, we first specified the frequency range of the objective lens (OL2) based on its NA where (−*2π/λ*) × NA_obj_ < *k*_*x*_ < (*2π/λ*) × NA_obj_, (−*2π/λ*) × NA_obj_ < *k*_*y*_ < (*2π/λ*) × NA_obj_ and *k*_*z*_^2^ + *k*_*x*_^2^ + *k*_*y*_^2^ = (*2π/λ*)^*2*^. In Fig. 2A, the frequency range is the area on the dome projected by the collection cone of OL2. Because of the angled alignment between OL3 and OL4, only partial frequency range at the back pupil of OL2 can be collected, illustrated in Fig. 2A as the overlapping dome area between two offset cones representing the collection ranges of OL2 and OL4. Thus, an additional constrain was applied to further limit the collection range in the back pupil of OL2: (−*2π/λ*) × NA_obj_ < *k*_*y*_ *<* *m* × (−*2π/λ*) × NA_obj_, where *m* is about 0.5 (Supplemental Fig. 3).

After the spatial frequency range was identified and given the value of ones, a 3D FFT was performed, and the corresponding point spread function (PSF) for illumination and the emission collection were obtained by taking the absolute values. The final system resolution for fluorescence imaging is the multiplication of the PSFs by illumination and collection. The values of NA_obj_, *k*_*offset*_ were adjusted to for different numerical apertures as summarized in Table 1.

**Table 1:** Summary of NA_obj_ and *k*_*offset*_ set up.

**NA**_**obj**_	**0.10**	**0.20**	**0.30**	**0.50**	**0.75**	**0.95**
*k*_*offset*_	0.05*k*	0.10*k*	0.20*k*	0.40*k*	0.65*k*	0.85*k*

### Preparation of GFP expressing human intestinal organoids (HIOs) derived from iPSC

Genetically modified iPSC expressing ubiquitous eGFP were used to differentiate human intestinal organoids (HIOs). Following the protocol described by Spence *et al*.^36^, we first incubated highly confluent iPSC with 100 ng/μL of Activin A (R&D; 338-AC-010) during three days to promote cell differentiation into definitive endoderm. Subsequently, we pushed the cells towards mid- and hindgut specification with 2 μM of the Glycogen synthase kinase 3 (GSK-3α/β) inhibitor, CHIR-99021 (Stemgent; 04–0004), and 500 ng/mL of human fibroblast growth factor 4 (hFGF4) (R&D; 235-F4-025) during five days. At this point, 3D structures emerged from the cell monolayer as floating spheroids expressing the intestinal marker CDX2. These spheroids were 3D cultured in Matrigel matrix (Corning; 354234) and incubated with 2 μM of HEPES buffer, 1% B27 supplement (Invitrogen; 12587-010), 500 ng/mL of Rspondin1 (R&D, 4645-RS-025), 100 ng/mL of Noggin (R&D; 6057-NG-025) and human epidermal growth factor (hEGF) (R&D; 236-EG-200) as pro-intestinal growth factors. After seven days, the organoids were split and re-plated again on plastic coverslips for imaging.

### Retinal vascular staining

Mice were euthanized and the eyes were enucleated. The eye balls were fixed in 4% Paraformaldehyde (PFA) for 15 mins, then transferred to 2% PBS for 20 mins. The anterior chamber, ocular lens, and vitreous, were carefully removed; the retina was then carefully removed from the choroid and flat-mounted on a clean petri dish. Excessive PBS was removed by a piece of paper, and −20 °C methanol was drop by drop applied on top of the retina to facilitate the staining. Retinas were moved to 2 ml tubes in cold methanol for at least 20 mins. After methanol fixation, retinas were washed several times with PBS, incubated with 100 ul perm/block solution (PBS + 0.3% Triton + 0.2% BSA) + 5% of goat serum per retina for one hour on a gently shaking rocker. After incubation, the perm/block solution was removed and each retina was incubated with 100 μl Alexa-488 conjugated isolectin-B4 solution (1/100 dilution in perm/block solution) overnight in 4 °C on a gentle rocker in the dark. After the overnight staining, retinas were washed 4 × 30 mins in PBS + 0.3% Triton (PBSTX). Then the retinas were mounted on glass slides with mounting media for imaging.

### Image processing and co-registration

For the OCT images, a horizontal surface would appear tilted in the slow scanning *y’* direction due to the angled illumination. This tilt was first corrected by shifting the *z’* dimension at each *y’* location. For the fluorescence images, two steps were taken to adjust the distortion of the image before the co-registration with OCT. First, the cross sectional image in *x’-z’* was warped to transfer the image shapes from trapezoid to rectangle, to co-register with OCT B-scan images. Then the z dimension was resampled at each *y’* location to maintain a constant and linear scale of depth. The volumetric co-registration between OCT and fluoresce images was slightly adjusted for each experiment to account for the different sample positioning. For biological samples, the refractive index of 1.38 was assumed in OCT reconstruction. An example of the image processing is provided in Supplemental Fig. 4. We should note that all the 3D rendering throughout the manuscript automatically assigned *x’*, *y’* and *z’* as mutually orthogonal coordinates, while physically *z’* is tiled due to the oblique illumination as illustrated in Fig. 1C.

## Results

The concept of oblique illumination with a single objective lens is illustrated in Fig. 2A, showing the 3D reciprocal Fourier domain in *k*_*x*_, *k*_*y*_, *k*_*z*_ at the back pupil plane of the objective lens (OL2). The dome represents all the spatial frequency at a particular *k* = *2π/λ* in the backward direction. Conventional LSM aligns the optical axis of the laser beam (at neutral scanning position) along with that of the objective lens. Here in OSLM, the laser incidence is offset in Fourier domain from *k*_*z*_ axis as in OPM and SCAPE (*i.e*. blue area in Fig. 2A), so that the laser is focused obliquely after the objective lens. In the detection of the fluorescence emission, the angled imaging system by OL4 (green cone) only collects partial spatial frequency range of OL2 (light pink cone). The overlapping area projected on the spherical dome ultimately defines the diffraction-limited resolution of the imaging system.

### Characterization of the system resolutions

With the Fourier domain model of the OSLM, we can characterize the theoretical resolution in 3D by a Fourier transform (see methods for detail information),

1 $$I({\bf{r}})=\int f({\bf{k}})\,{e}^{j{\bf{k}}{\bf{r}}}d{\bf{k}},$$

where *I*(**r**) is light field at a spatial location **r**, and *f*(**k**) is the reciprocal Fourier domain distribution in **k**. Figure 2B shows the examples of the point spread function (PSF) of an oblique illumination by an effective NA = 0.04 at wavelength λ = 0.5 μm. We created the oblique illumination by offsetting the optical axis of the laser, which is equivalent by shifting the frequency range by *k*_*y,offset*_ = 0.4*k*, as illustrated in Fig. 2B. Because of the small NA, the illumination beam profile is fairly consistent within ±150 μm depth range beyond which the beam starts to defocus.

For fluorescence collection, only a portion of the spatial frequency can be detected due to the angled alignment of the final imaging system (Fig. 2A). The frequency range for collection is verified at the back pupil plane of OL4 by using a thin fluorescein solution sandwiched between two coverslips (Supplemental Fig. 3). The corresponding PSF for fluorescence emission is shown in Fig. 2C. By multiplying the PSF of illumination and collection, the final 3D PSF from fluorescence emission can be generated in Fig. 2D. We then characterized the resolutions over a range of NA of the objective lens (OL2) in Fig. 2E, with the same illumination NA. Because of the relative angle between the illumination and collection, the axial resolution reduced rapidly by ~10 fold from NA = 0.1 to 0.3.

The resolution for OCT is relatively straightforward since only the incident beam is relevant. The beam width defines the lateral resolution, which is estimated at ~7 μm. The depth resolution is determined by the OCT spectral coverage. A Gaussian spectral window centered at 562 nm with 30 nm FWHM was applied to reshape the spectrum to improve the image quality. Thus, the depth resolution in OCT is estimated to be ~3.5 μm in water.

**Figure 3:**
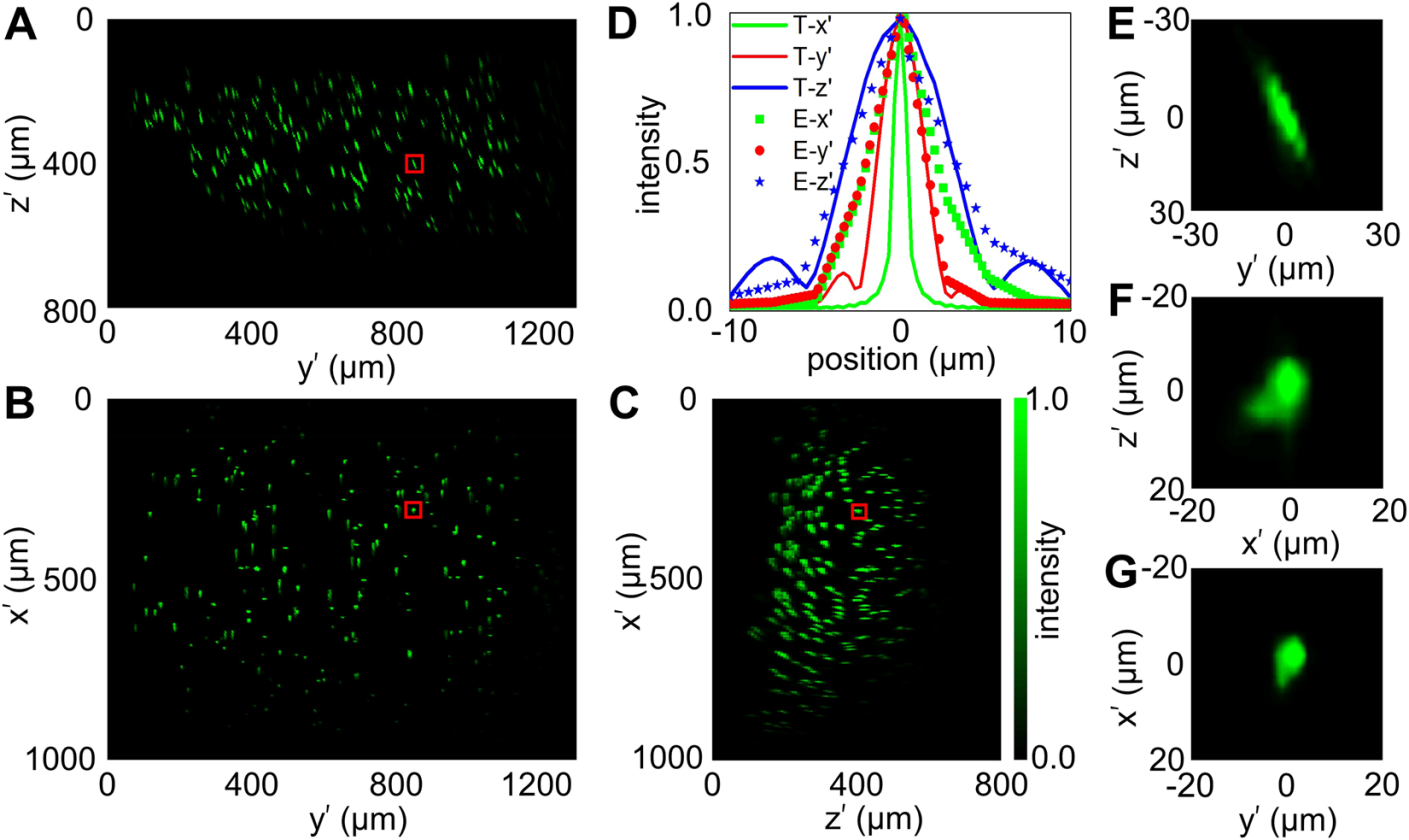
Experimental resolution characterization of the fluorescence imaging in OSLM. (**A–C**) The maximum intensity projections of 1 μm fluorescent beads in 0.5% Agarose gel. (**D**) The intensity profile cross the center of a representative 1 μm fluorescent microsphere red-squared in (**A–C**), as *E*-*x’*, *E*-*y’* and *E*-*z’*. The theoretical PSF is plotted in the same panel for comparison, as *T-x’*, *T-y’*, and *T-z’*. (**E–G**) Zoomed in images of the 1 μm bead in *y’-z’* (**E**), *x’-z’* (**F**), and *x’-y’* (**G**) sections.

Next, we experimentally characterized the fluorescence PSF by imaging fluorescent microspheres with 1 micron diameter. The microspheres was immobilized within a 0.5% agarose gel and the example of the images are shown in Fig. 3. The FOV is about 0.8 mm by 1 mm by 0.5 mm in *x’*, *y’*, *z’* directions. Note that we used the subscription of prime to separate from conventional notations for Cartesian coordinate system, because *z’* is along the direction of the oblique laser illumination (Fig. 1C). The zoomed-in images of one microsphere in the center of the FOV are displayed in Fig. 3E–G. The resolutions are characterized by the FWHMs of the fluorescence intensity cross the center of the sphere, which are measured around 3.6 μm, 4.2 μm, and 6.5 μm in *x’*, *y’*, *z’* directions, respectively. The theoretical PSF is plotted in the same figure for comparison. The FWHM along the longest axis of the PSF in the simulation (Fig. 2D) and experiment (Fig. 3E) is ~5.1 and ~10.7 µm, respectively. The experimental values are larger than the simulation due to the optical aberration from the relay optics and the scatter light from the two parabolic mirrors in the scanning unit.

### Phantom images of fluorescent and plain microspheres

**Figure 4:**
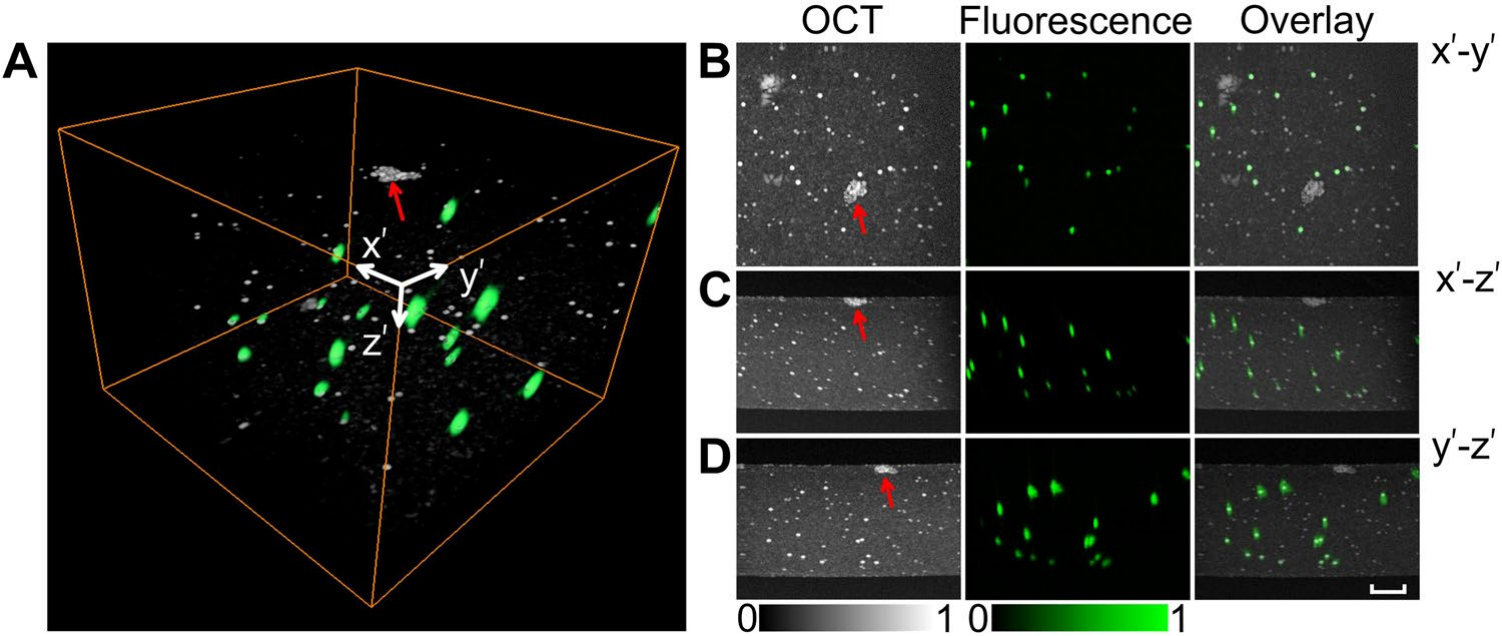
Phantom images of 2 μm plain microspheres and 3 μm fluorescent microspheres in Agarose gel. (**A**) The merged three-dimension OSLM images with 2 μm beads and 3 μm beads. (**B–D**) The maximum intensity projections in *x’-y’*, *x’-z’*, and *y’-z’* sections for OCT, fluorescence, and the overlay, respectively. OCT images shows all the scattering subjects including the weakly scattering gel, and a dust particle (red arrows), while fluorescence specifically labeled the 3 μm beads. Bar 100 μm. See the fly-through of the depth sections in the supplemental movie.

To demonstrate the capability of simultaneous 3D structure and fluorescence imaging by OSLM, we imaged a mixture of fluorescent (FluoresbriteTM plain YG, 3 μm in diameter, Polysciences Inc.) and non-fluorescent (Latex microsphere suspensions, mean diameter 2 μm, Thermo scientific) microspheres sealed in Agarose gel (Fig. 4A). OCT is sensitive to refractive index fluctuations, and thus can image any structural heterogeneities. This is evidenced by both types of spheres appearing in OCT images with different sizes and brightness, as well as the agarose gel background, and a dust on the surface of the gel. On the other hand, the emission of fluorophore provided the molecular specification so that only the fluorescent microspheres appeared in the fluorescence images. The overlaid images in three projections show their distinct features in both modalities, and the co-registration in 3D (Fig. 4B–D).

### ***In vitro*** intestinal organoids derived from GFP expressing induced pluripotent stems cells (iPSC)

**Figure 5:**
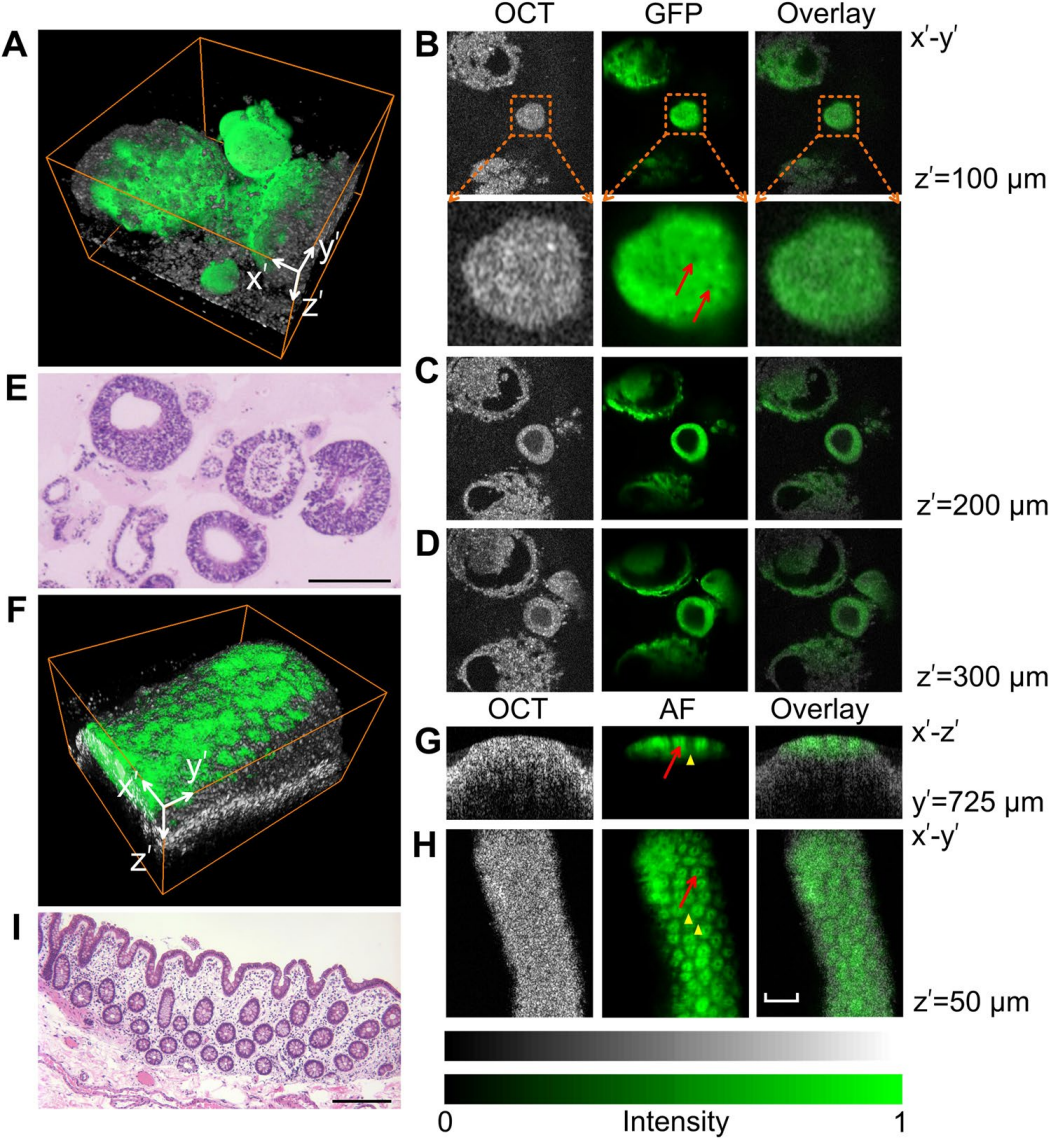
*In vitro* HIOs differentiated from iPSC (**A**–**E**), and *ex vivo* rat colonic mucosa (**F**–**I**) OSLM imaging. (**A**) 3D overlay image of OCT and GFP fluorescence. The field of view in fluorescence is slightly smaller than OCT, so the fluorescence signal fades at the far ends of *x’* axis. (**B**–**D**) Images at different depth sections through organoids in OCT, GFP, and their overlay. In panel B, the 4x zoomed in images are shown below the main images. Red arrows point to the cell nuclei in the GFP image. (**E**) Histology of HIOs. (**F**) A similar image as panel A where the fluorescence signal is autofluorescence (AF) from FAD and collagen. (**G**–**H**) Images of a cross-sectional plane and a depth section in OCT, AF, and their overlay. Red arrows point to the crypts, and yellow triangles point to the AF background from lamina propria. (**I**) Histology of rat colon. For panel F-H, data was acquired using 25 kHz OCT A-line rate, and 70 ms camera exposure time. See the fly-through of the depth sections in A and cross sections in B in the supplemental movies. Bar 200 µm.

Figure 5A–E shows images of *in vitro* human iPSC-derived intestinal organoids (HIOs) from a healthy individual. Intestinal organoids formed a structure with the intestinal epithelium bordering a central lumen, mimicking the natural anatomy of the digestive tract. Remarkably, mature intestinal organoids can faithfully recapitulate the *in vivo* tissue architecture and contain the full complement of stem, progenitor and differentiated cell types, which makes iPSC-derived organoids a valuable tool to study human diseases^37^. Figure 5A is the overlay 3D rendering of the GFP-expressing HIOs, and Fig. 5B–D are OCT, fluorescence imaging and their overlay images at different depth sections. At the topical section of the organoid in Fig. 5B, there is a lack of cellular or sub-cellular contrast in OCT because all the structures gave rise to OCT signal. In contrast, we can identify the cell nuclei and recognize individual cells in the zoomed in fluorescence images, presumably due to higher GFP expression in cytoplasm rather than in nuclei. In the cross sections through the center of the organoids (Fig. 5C,D), the inside lumen and the bordering epithelium can be clearly visualized and matched, as compared to the histological image in Fig. 5E.

### *Ex vivo* autofluorescence imaging of rat colonic mucosa

We also demonstrated that the intrinsic fluorescence can be used as the label-free molecular contrast from an *ex vivo* sample of rat colonic mucosa as in Fig. 5F–I. While OCT provided the overall morphology of mucosa, the intracellular autofluorescence images highlighted the crypts as the cylindrical structures composed of epithelial cells. The anatomical structure is exemplified in the histology (Fig. 5I). Within epithelial cells, the autofluorescence is predominantly from Flavin group under 420–470 nm excitation in our current setup^38, 39^. A weak background fluorescence can also be detected from the lamina propria supporting the epithelium, largely composed of collagen networks. The penetration depth in autofluorescence 3D imaging was less than OCT, due to the strong attenuation of the excitation light.

### *Ex vivo* imaging of 3D retinal vasculature

**Figure 6:**
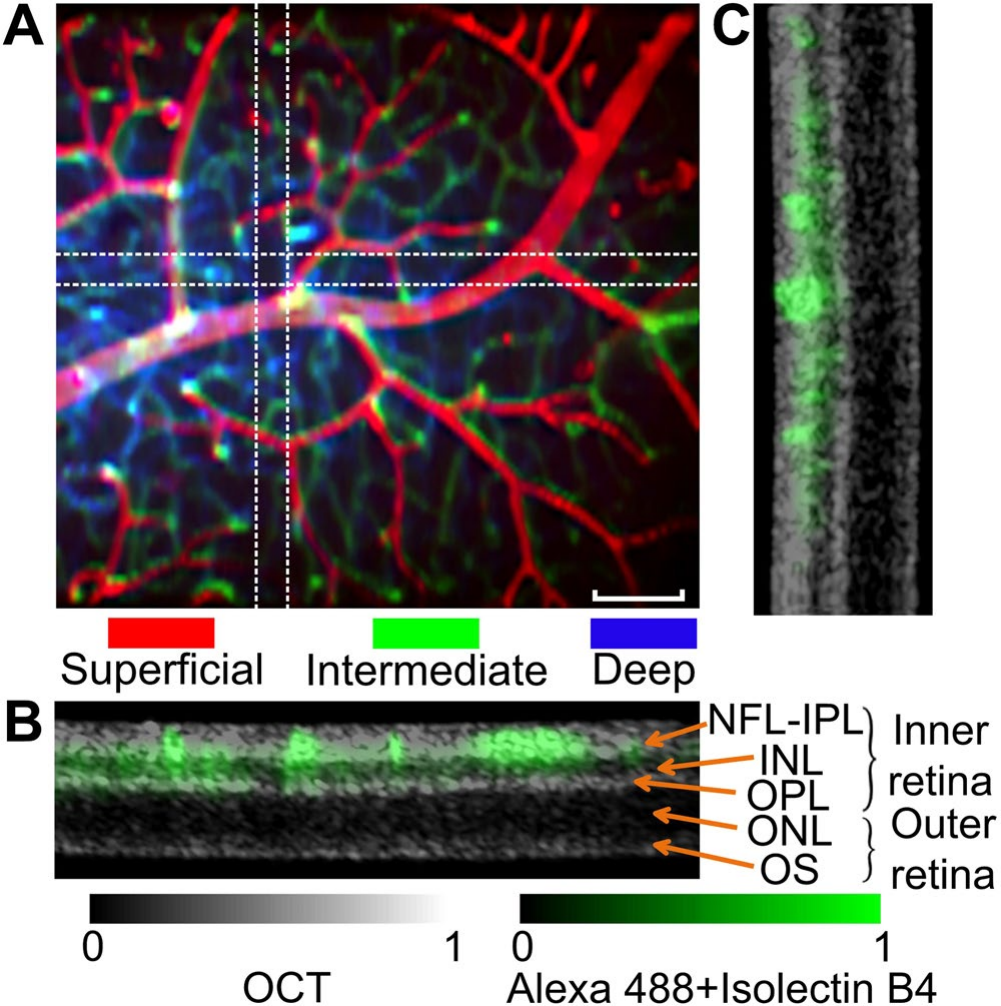
OSLM images of *ex vivo* mouse retina. (**A**) Depth-encoded retinal vasculature in three depths (superficial, intermediate, and deep). Three layers are taken from about 0–20 μm, 40–50 μm, and 70–90 μm from the retina surface, respectively. The endothelium is labeled by Alexa 488 conjugated isolectin B4 antibody. (**B**,**C**) The overlay images of OCT and fluorescence at two orthogonal cross-sections. The retinal circulation is located within inner retina. NFL: neural fibre layer; IPL: inner plexiform layer; INL: inner nuclear layer; OPL: outer plexiform layer; ONL: outer nuclear layer; OS: outer segment of photoreceptors. See the fly-through of cross sections in the supplemental move. Bar 100 µm.

To demonstrate the potential applications of OSLM in the ophthalmic imaging, Fig. 6 shows a depth-resolved image of *ex vivo* retinal vasculature. After the dissection of mouse retina, the endothelium was stained by isolectin-B4 and the three layers of the retinal circulation can be discriminated by their individual depth (Fig. 6A). The three layers were taken at around NFL, IPL, and OPL. In the simultaneous OCT images, the anatomical layer structure can be seen as the typical OCT retinal images. As the overlaid image shows, the retinal circulation is located within inner retina, and the outer retina is avascular (Fig. 6B,C).

## Discussion

We described a multimodal system termed OSLM capable of high resolution macroscopic volumetric imaging for simultaneously structural and molecular imaging. The oblique illumination scheme with a single objective lens circumvents the complexity of two orthogonal lenses and mechanical translations in conventional light sheet fluorescence microscopy. Instead of 2D light sheet illumination, OSLM uses a 1D scanning laser and thus allows the seamless integration of OCT. Therefore, structural and molecular volumetric imaging can be simultaneously achieved over a macroscopic volume with high resolution by using just one 2D raster scan. We demonstrated three independent mechanisms to provide the molecular specificity: intrinsically expressed fluorescent protein (GFP organoids as in Fig. 5), intrinsic autofluorescence (FAD in colon mucosa in Fig. 5) and exogenous antibody-conjugated dyes (Alexa conjugated isolectin B4 in Fig. 6). Other contrast mechanisms based on laser scanning microscopy can be potentially incorporated in OSLM to expand its applications, such as Raman/stimulated Raman scattering^40^, coherent anti-Stokes Raman scattering^41, 42^, second harmonic generation^43^, and others.

The major limitation of the current system is the speed. The CCD camera that we used for OSLM requires 30 ms read out time after each exposure, which only allows 40% duty cycle with 20 ms exposure. The speed of imaging can be drastically improved by using high speed sCMOS camera as previously used in OPM and SCAPE. Meanwhile, the image of OCT has been rapidly improved in recent years. Conventional Fourier domain OCT can operate at 312 kHz^44^ and 500 kHz^45^ A-line rate *in vivo*, and the state-of-the-art swept source OCT can achieve over 1 MHz A-line rate^46^. Assuming the volumetric imaging with a 200 × 200 pixel density in *x’-y’* plane, the above OCT Aline rate would translate to 6.25, 12.5, 25 vps, which is similar to the reported imaging speed in SCAPE^13^. Another limitation of the current system is that the fluorescence imaging has a smaller field of view and lower depth resolution than OCT, due to the size of relay optics and the relatively small NA of the objective lens (OL2), respectively. This will be overcome by optimizing the optical components and choosing an objective lens with higher NA.

One major motivation behind this work is to seamlessly merge OCT structural imaging with fluorescence imaging in 3D. The benefit of structural imaging by OCT is not only to co-register molecular imaging to tissue morphology, but also offer excellent sensitivity to characterize the native structural changes. OCT relies on the elastic light scattering which is ultimately determined by the refractive index (RI) fluctuation. The RI in biological tissue is proportional to mass density across the major macromolecular compositions^47, 48^, making elastic light scattering an excellent measure of tissue structures. More importantly, the sensitivity of elastic light scattering to structural alteration can be at the scale of several tens of nanometers, beyond the resolution limit of conventional microscopy^16, 49^. We have also demonstrated a method using OCT spectroscopic analysis to sense structural changes down to ~40 nm^15^, without the need to resolve the detailed nanoarchitecture. This provides a powerful tool to characterize and measure the local structural context, and complement the molecule-specific fluorescence imaging.

Granted that the imaging speed will be significantly improved, there are several applications that can potentially benefit from high speed OSLM. For example, the vascular dysfunction has major implications in a broad range of diseases, including cancers, diabetes, cardiovascular diseases and neurodegenerative diseases. OCT is an excellent tool to study vascular functions by quantifying blood flow^17, 18^, capillary flux^50^, blood oxygenation^19, 20^ and microangiography^21, 22^. Combining a high speed OSLM, the 3D dynamics and interaction of different cell types could be visualized in real time with specific fluorescence reporters, and how they impact hemodynamics can be studied. Another example is the ophthalmic imaging, OCT has been the standard of care in eye care for preventing blindness. Application of OSLM with the ocular lens would allow a large FOV 3D fluorescence imaging in retina, with the simultaneous OCT structural imaging as exemplified in Fig. 6. When fully dilated, the ocular lens has the effective NA around 0.5 for rodents and 0.2 for human. Based on the theoretical prediction in Fig. 2, the depth resolution would be around 6 μm and 30 μm, respectively, which would be sufficient to resolve the major anatomical layers *in vivo*. Beyond the *in vivo* applications, the imaging of iPSC HIOs presents an example of *in vitro* applications. It has been reported that the scattering contrast from OCT can be a sensitive markers for early carcinogenesis before the microscopic structural changes^51^. Using patient’s specific iPSC organoids, OSLM can potentially produce combined optical markers to study early malignancy transformation for personalized cancer prevention and treatment.

## Electronic supplementary material

Supplemental materials

fly-through movie for microspheres phantom

fly-through movie for iPSC HIOs

fly-through movie for colon mucosa

fly-through movie for mouse retina
