# Supplementary material for: Oblique scanning laser microscopy for simultaneously volumetric structural and molecular imaging using only one raster scan
Source: Sci Rep. 2017 Aug 17;7:8591. doi: 10.1038/s41598-017-08822-0 (PMC5561209; doi:10.1038/s41598-017-08822-0)
Supplement: Supplementary file 1 — Supplemental materials [file 41598_2017_8822_MOESM1_ESM.doc]

Oblique scanning laser microscopy for simultaneously volumetric structural and molecular imaging using only one raster scan

Lei Zhang^1^, Amalia Capilla^1,2^, Weiye Song^1^, Gustavo Mostoslavsky^1,2^, Ji Yi^1,2,3^*

1. Department of Medicine, Boston University School of Medicine, Boston, MA 02118
2. Center of Regenerative Medicine, Boston University, Boston, MA 02118
3. Boston University Photonics Center, Boston, MA 02215

*Corresponding author: [jiyi@bu.edu](mailto:jiyi@bu.edu)

Zemax simulation on oblique laser illumination

The imaging system is simulated by using the commercial software of Zemax-OpticStudio16. Figure 1A shows the optical path of the illumination. The objective lens (OL2) is substituted by an ideal thin lens. The focal length of the thin lens in the model is estimated to be 8.6 mm, given a 10 mm back pupil diameter and a 0.5 NA of the lens (UplanFL N 20×/0.5, Olympus). The scanning laser was created by changing the angle of the galvanometer mirror (GM2). The *x-z* and *y-z* longitudinal cross sections of the oblique laser focus are shown in Fig. 1B and Fig. 1C, respectively. The ~26˚ angle of the illumination is created in *y-z* plane by a 4 mm offset of the objective lens.


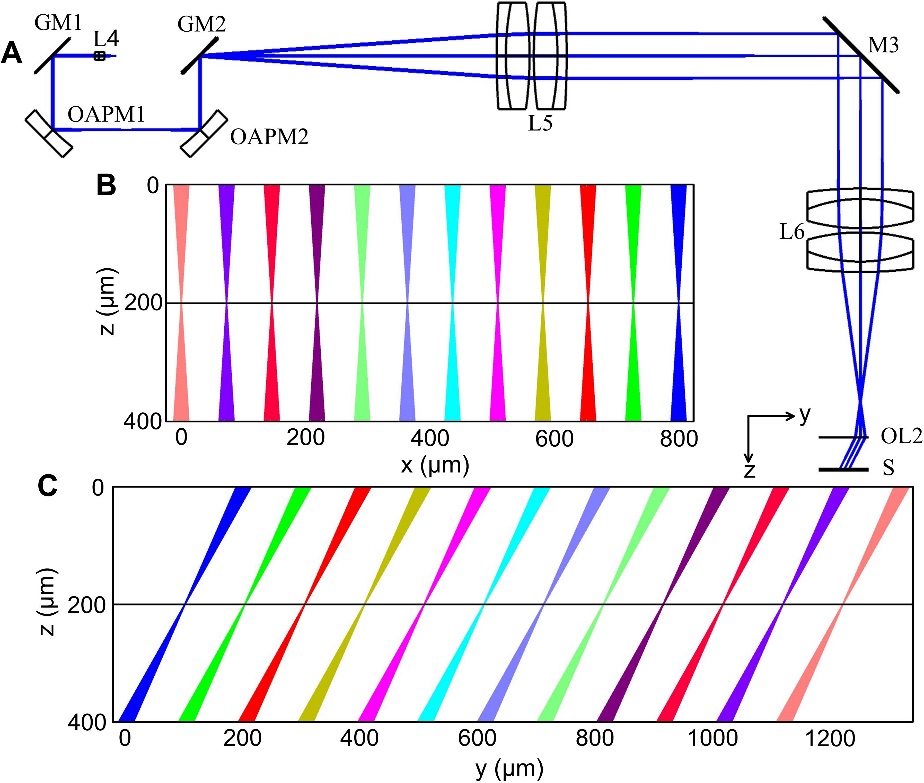


Figure 1. The Zemax-OpticStudio simulation for the illumination of the OSLM system. (A) The optical path; (B) The *x-z* and (C) *y-z* longitudinal cross sections of the volume illuminated by the oblique scanning laser.


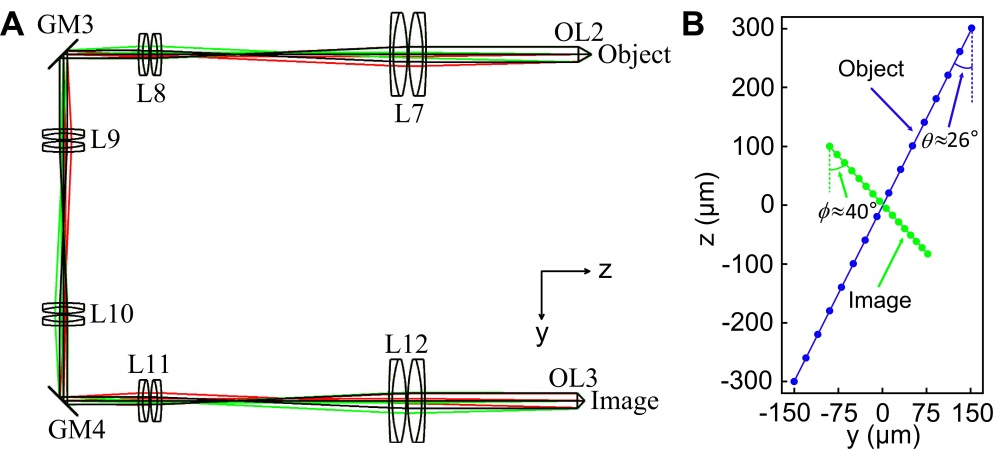


Figure 2. The Zemax-OpticStudio simulation for the fluorescence light. (A) The illumination optical path from the sample to the OL3. (B) The point sources along the oblique laser illumination in *y-z* plane in an angle of 26º, and their corresponding images after OL3. No scanning or de-scanning was introduced in the simulation.

Next, we simulated the image of the oblique laser illumination after OL3. Since the relay optics (L7 to L12) between OL2 and OL3 collectively has no magnification, the magnification from the sample plane in front of OL2 to the image plane after OL3 is calculated to be 2/3 in transverse plane determined by the NAs of OL2 and OL3 (0.5 and 0.75), and ~(2/3)^2^ =4/9 in axial direction [1]. Thus, the 26˚ angle of the oblique illumination will be enlarged to ~36˚, in a relation of

(1)

where M is the imaging magnification, *θ* and *ϕ* are the angle of oblique laser illumination and its conjugate image, with respect to the optical axis of the system. Figure 2A shows the partial optical path for the fluorescence detection. In the simulation, the positions of the de-scanning mirrors are fixed at 45 degree with respect to the optical axis. We set up a series of point sources along the oblique laser illumination in an angle of 26˚, and the corresponding images of all the points determines the angle of ~40˚ of the image after OL3 (Fig. 2B). The magnification in simulation is ~0.58 and ~0.31, in transverse and axial direction respectively.

Fluorescence detection range in *en face* view of the Fourier space

We tested the fluorescence detection range in the Fourier domain using a thin layer of fluorescein solution sandwiched between two cover slips. The last focusing lens (L13) in front of the CCD camera was removed, and the spatial frequency range of fluorescence detection can be imaged. Figure 3 shows the image after OL4 from the thin layer of fluorescein. The large pink circle represents the detection limit of OL2, and the dashed green line represents the detection limit of OL4 after OL3. The detection range roughly took up about one quarter of the *k_y_* range from OL2, and filled up about half of the OL4’s back pupil plane.


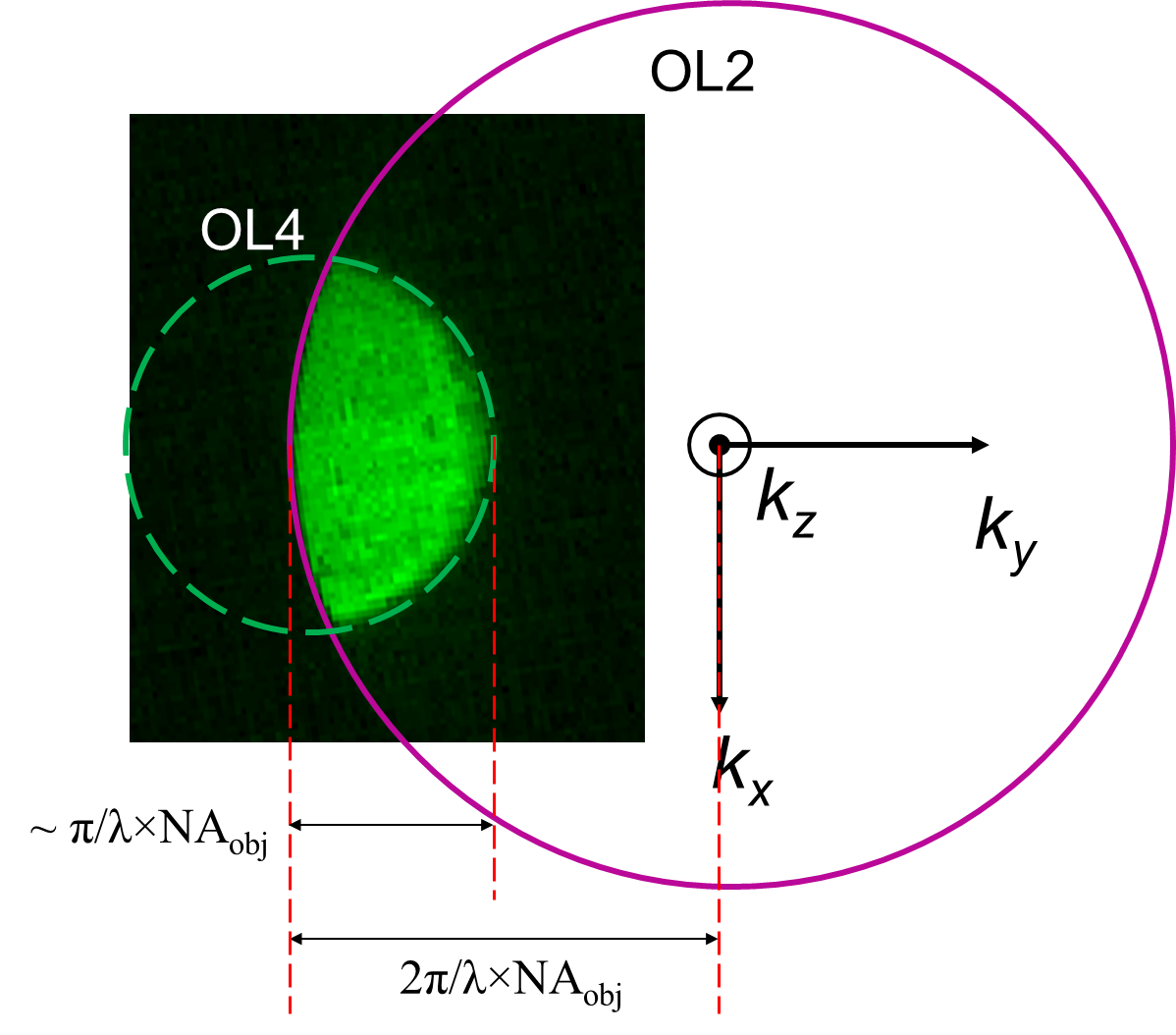


Figure 3. The fluorescence detection range in *en face* view of the 3D Fourier space. The large pink circle represents the detection limit of OL2, and the dashed green line represents the detection limit of OL4 after OL3. The fluorescence image was taken by the CCD camera after OL4 from a thin fluorescein solution. The image was false-colored in green to represent the green fluorescence. NA_obj_ is the numerical aperture of OL2.

Image processing and co-registration using four-layer fluorescein solution mixed with 0.08 μm beads

We tested the image processing and co-registration on four-layer fluorescein solution mixed with 0.08 μm beads. The four-layer structure was formed by sandwiching the solution between a glass slide and four pieces of coverslips. The horizontal surface appeared tilted in the *y’-z’* plane in OCT due to the oblique illumination, as shown in the original column of Fig. 4B. The elements in the *z’* direction were circularly shifted to make the surface horizontal. For the fluorescence figures, two corrections were made before co-registering with OCT images. First, the elements in *x’-z’* plane were warped to transfer a trapezoid shape to rectangle as shown in Fig. 4C. Then, interpolations were operated at each column in *x’-z’* plane for a linear depth scale (Fig. 4C), and in *y’-z’* section for equal distance between four layers (Fig. 4D). After these processes, the OCT and fluorescence images are co-registered in 3D as shown in Fig. 4A. These image processing algorithms and parameters were then used to process all the tissue figures in the main text. For different samples, the parameters was slightly adjust to account for different sample positions.


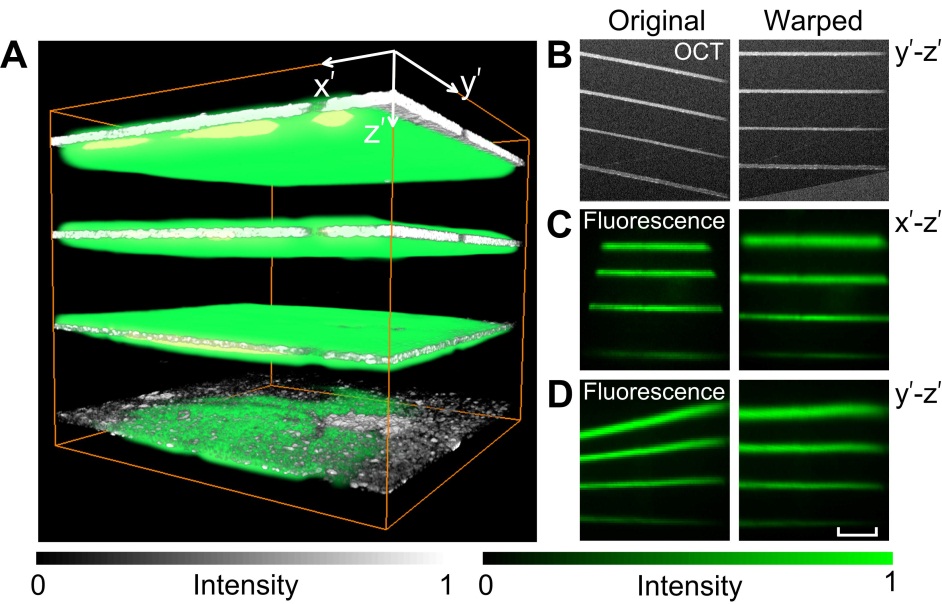


Figure 4. The images for the four-layer fluorescein solution mixed with 0.08 μm beads. (A) 3D images of OCT and fluorescence. (B) The *y’-z’* section of the OCT image. (C-D) The *x’-z’* and *y’-z’* sections of the fluorescence image. The slow scan axis is along *y’*. Bar 200 μm.

System components

Table 1. The information of the system components.

| Component | Modal | Manufacturer |
| --- | --- | --- |
| SL | SuperK EXTREME EXU-6 | NKT Photonics |
| F1 | DMLP650R | Thorlabs |
| F2 | ZT514/1064rpc | Chroma |
| F3 | ET512/20&MF525-39 | Chroma&Thorlabs |
| BT1/BT2 | BTC30 | Thorlabs |
| PBS | CM1-PBS251 | Thorlabs |
| DM | BBD1-E02 | Thorlabs |
| P1/P2 | PS853 | Thorlabs |
| OL1 | DIN 20 0.4 | Edmund Optics |
| OL2 | UplanFL N 20×/0.5 | Olympus |
| OL3 | UplanSApo 20×/0.75 | Olympus |
| OL4 | UplanFL N 10×/0.3 | Olympus |
| OL5 | Plan N 4×/0.1 | Olympus |
| OL6 | UplanF1 10×/0.3 | Olympus |
| OFC | TW560R5A2 | Thorlabs |
| L1 | HPUCO-23A-400/700-S-50AC | OZ Optics |
| L2 | Multi-elements lens *f*=150mm | JML optics |
| L3 | HPUCO-23A-400/700-PSM-10AC | OZ Optics |
| L4 | HPUCO-23A-400/700-PSM-4.5AC | OZ Optics |
| L5 | AC254-300-A×2 | Thorlabs |
| L6 | AC254-150-A×2 | Thorlabs |
| L7/L12 | AC508-200-A×2 | Thorlabs |
| L8/L9/L10/L11 | AC254-100-A×2 | Thorlabs |
| L13 | HR F2.8/50mm | Navitar |
| L14 | AC508-250-A | Thorlabs |
| VNDF | NDC-50C-4M-A | Thorlabs |
| DC | WG11010 | Thorlabs |
| GM1/GM2/GM3/GM4 | GVS201 | Thorlabs |
| G | 1800 lp/mm transmission grating | Wasatch |
| AS | VA100C | Thorlabs |
| OAPM1/OAPM2 | MPD129-p01 | Thorlabs |
| Camera | Pco.pixelfly usb | PCO |
| PMT | H11459-01 | Hamamatsu |
| LSM | Sprint spl2048-140km | Basler |

Reference:

[1] Botcherby, Edward J., et al. "An optical technique for remote focusing in microscopy." *Optics Communications* 281.4 (2008): 880-887.
